# Comparative Evaluation of Piezosurgery Versus Conventional Surgical Implant Placement and Impact on Implant Stability, Bone Density, and Patient Comfort: A Randomized Clinical Study

**DOI:** 10.1155/ijod/8187491

**Published:** 2025-11-27

**Authors:** Manish Rathi, Dinesh Yadav, Sangeeta Singh, Anil Kumar Jha

**Affiliations:** ^1^Department of Periodontology, Army Dental Centre (R&R), New Delhi, India; ^2^Army Dental Center (R&R), New Delhi, India; ^3^Examiner in Periodontics, Royal College of Surgeons of Edinburgh, UK; ^4^Department of Periodontology, Saraswati Dental College, Lucknow, Uttar Pradesh, India

**Keywords:** bone density, dental implants, implant stability, minimally invasive surgery, piezoelectric surgery

## Abstract

**Purpose:**

This study aimed to comparatively evaluate piezoelectric surgery and conventional surgery for dental implant site preparation, focusing on bone density, implant stability, and peri-implant marginal bone loss, to contribute valuable insights into optimizing dental implant procedures for improved patient care and treatment outcomes.

**Material and Methods:**

In this randomized controlled clinical trial with a split-mouth design, 30 patients with a mean age of 35.7 (standard deviation [SD] 7.1) with two edentulous sites, at least one of which was in the posterior mandibular region, were treated sequentially at two sites: Site A, where implant placement was conducted using piezoelectric surgery, and Site B, where conventional surgery was employed. Postsurgical evaluations were conducted at 6 and 9 months.

**Results:**

Significant differences were observed between the two techniques. Bone density was greater by 0.035 g/cm^2^ (*p*=0.001) in favor of piezoelectric surgery at 9 months, but not at 6 months. Although there were significant changes in stability within the groups, the difference in the change in implant stability at 6 months between piezoelectric surgery and the control site was not significant at 6 months, Site A (piezoelectric surgery) demonstrated significantly higher implant stability quotient (ISQ) values (mean = 76.7) than Site B (mean = 72.8; *p* ≤ 0.001). Patient discomfort was significantly greater in the control group than in the piezoelectric surgery group by 1.3 visual analog scale (VAS) (*p*-value < 0.001).

**Conclusion:**

Piezoelectric surgery demonstrated potential benefits over conventional surgery in terms of implant stability, postoperative discomfort, and bone density. These findings highlight the potential of piezoelectric surgery to enhance clinical outcomes and patient satisfaction in dental implant procedures. Further investigation into long-term implant survival rates and esthetic outcomes is warranted.

**Trial Registration:** ClinicalTrials.gov identifier: ISRCTN99951388; 16CNAHMMDS000002/ISRCTN99951388

## 1. Introduction

Over the past few decades, dentistry has experienced remarkable advancements propelled by technological innovations [1]. Among these breakthroughs, the introduction of dental implants stands as one of the most significant, offering restoration solutions for complete or partial edentulism, the loss of natural teeth [2]. Edentulism, the ultimate consequence of multifaceted processes encompassing biological factors such as caries, periodontal disease, and trauma, as well as nonbiological factors like access to care and treatment preferences, underscores the complexity of dental restoration [3].

Dental implantology has evolved into a discipline not only focused on restoring edentulous spaces but also enhancing the overall quality of life for patients [4]. Continuous progress and deepened understanding of tissue biology have greatly improved the predictability and longevity of dental implants as substitutes for natural teeth [5]. Central to the success of an implant is its relationship with host tissues, particularly with bone [6].

The stability response of dental implants after insertion via piezoelectric surgery is well characterized. Following surgery, the mean stability of implants produced with both conventional drills and piezoelectric devices decreased significantly from baseline for the first 21 days, and then gradually improved to approach baseline stability or higher. Few studies found that implant stability was much higher in the group that received piezoelectric surgery compared to other surgical procedures [7–9]. Another study concluded that there was an early increase in implant stability quotient (ISQ) value in piezoelectric surgical osteotomy, suggesting a shorter inflammatory resorption phase [10]. Two more studies found piezoelectric surgery to be a viable alternative for implant osteotomy with a comparable implant survival rate to conventional drilling [11, 12]. Piezoelectric surgical osteotomy was associated with short-term clinical benefits, leading to an early rise in ISQ values and thereby leading to shorter time for implant loading [13]. Three other studies found no significant difference in implant stability between piezoelectric surgery and other surgical methods [14–16]. Implants inserted using piezoelectric surgery were, on average, 2.57 ISQ more stable than implants inserted using other procedures. It should be noted that the ISQs of implants inserted with ordinary drills and piezoelectric tools gradually converged. The present study reconfirms these findings.

The interaction between implants and soft tissues is dynamic but may pose biocompatibility challenges, making direct implant-bone interaction more desirable for long-term success [17]. Achieving this involves primary mechanical interlocking of the bone during implant placement, followed by continuous deposition of lamellar bone and remodeling along the implant surface, a process known as osseointegration [18]. This process ensures rigid fixation of the implant material within the bone, clinically asymptomatic, and maintained during functional loading [1].

However, ensuring successful clinical outcomes poses challenges for clinicians, necessitating control over various factors such as implant surface characteristics, biocompatibility, bone anatomy, surgical techniques, loading conditions, and undisturbed healing periods [19]. Traditional surgical approaches, involving elevation of full-thickness flaps and the use of rotary instruments like round burs and twisted drills, may have drawbacks, including heat generation leading to bone necrosis, excessive bleeding, neurovascular damage risks, and compromised esthetics [20].

Minimally invasive surgical techniques have gained prominence in overcoming these limitations. One such innovation is piezoelectric surgery, introduced by Vercelotti, which utilizes piezoelectric materials to cut mineralized tissues such as bone with precision and minimal damage to surrounding soft tissues. Piezoelectric surgery is based on the principle of piezoelectricity, which generates ultrasonic oscillations to facilitate precise cutting.

There is no consensus on patient-reported pain with piezoelectric surgery, with most studies showing less pain than conventional methods, while others indicate the opposite. Piezoelectric surgery for maxillary canine retraction caused greater pain compared to other methods during the first treatment week [21]. Piezocision-assisted orthodontic therapy led to more pain than traditional treatment, despite comparable painkiller intake [22]. For mandibular third molar extractions, piezoelectric surgery showed no significant difference in pain versus conventional techniques [23]. However, piezoelectric surgery resulted in less pain compared to conventional osteotomy for unerupted mandibular third molars in children [24] and impacted mandibular third molar surgeries [25]. Studies found reduced patient discomfort with piezoelectric surgery compared to standard drilling for implant preparation [26] and osseous resective surgery [27]. Simple tooth extractions with piezoelectric surgery caused less pain than traditional methods [28]. Given pain assessment's subjective nature and minimal observed differences, the disparity between traditional methods and piezoelectric surgery in pain perception appears negligible. There is no consensus on patient-reported pain in response to piezoelectric surgery, with most studies suggesting that it results in less pain than conventional methods, while others suggest the opposite. Piezoelectric surgery to assist in maxillary canine retraction resulted in greater patient-perceived pain compared to other methods during the first week of treatment [21]. Piezoelectric surgery used to assist with upper canine retraction also resulted in greater pain compared to laser-assisted flapless corticotomy (LAFC). In yet another study, despite comparable levels of painkiller intake and excellent patient satisfaction with the treatment length, piezocision-assisted orthodontic therapy led in more patient-perceived pain than traditional orthodontic treatment [22]. Piezoelectric surgery to elevate the surgical flap, perform osteotomy, and carry out odontosection resulted in no significant difference in patient-perceived pain compared to conventional techniques during mandibular third molar extractions [23]. On the other hand, piezoelectric surgery to extract unerupted mandibular third molars in children resulted in less patient-perceived pain compared to conventional osteotomy [24]. Piezoelectric surgery used for osteotomy in impacted mandibular third molar surgeries resulted in less patient-perceived pain compared to conventional bur techniques [25]. Another study found that piezoelectric surgery reduced patient discomfort compared to standard drilling for implant site preparation [26]. Piezoelectric surgery caused somewhat less discomfort in patients than typical rotational drills for osseous resective surgery in the treatment of periodontitis [27]. Piezoelectric surgery used for simple tooth extractions resulted in less patient-perceived pain compared to traditional surgical methods [28]. The subjective nature of pain assessment, coupled with the minimal differences observed in the aforementioned studies, suggests that there is negligible disparity between traditional methods and piezoelectric surgery in terms of patient pain perception. In a recent study, Vignudelli et al. [29] conducted the first histomorphometric analysis on humans to compare bone healing between piezosurgery and conventional drilling, revealing biologically significant differences in the early stages of healing [29]. The research question of this study was to specifically investigate pain perception within our patient cohort to enhance the understanding of the patient experience with piezoelectric surgery for dental implants. The null hypothesis was that there is no difference in patient-reported outcome measures regarding pain perception between traditional and piezosurgical implant site preparation.

Further, this study aimed to comparatively evaluate piezoelectric surgery and conventional surgery in implant site preparation, focusing on implant stability, bone density, and patient pain perception. By exploring the long-term outcomes of these techniques, this research seeks to contribute novel and valuable insights into bone density and patient pain perception, allowing the optimization of dental implant procedures for improved patient care and treatment outcomes.

## 2. Materials and Methods

The present study was designed as a split-mouth randomized clinical trial and performed according to the Declaration of Helsinki and the International Conference on Harmonization (ICH) for Good Clinical Practice (GCP). The consort statement list was completed by the first author (Manish Rathi) and complied with the CONSORT guidelines/checklist. The protocol was registered in the ISRCTN registry and approved by the Institutional Ethical Committee of ADC (R&R), New Delhi (India), with Approval Number: 16CNAHMMDS000002. Enrollment of patients began in December 2016, and the 1-year observation period was finalized in October 2023.

### 2.1. Selection and Grouping of Subjects

**Figure 1:**
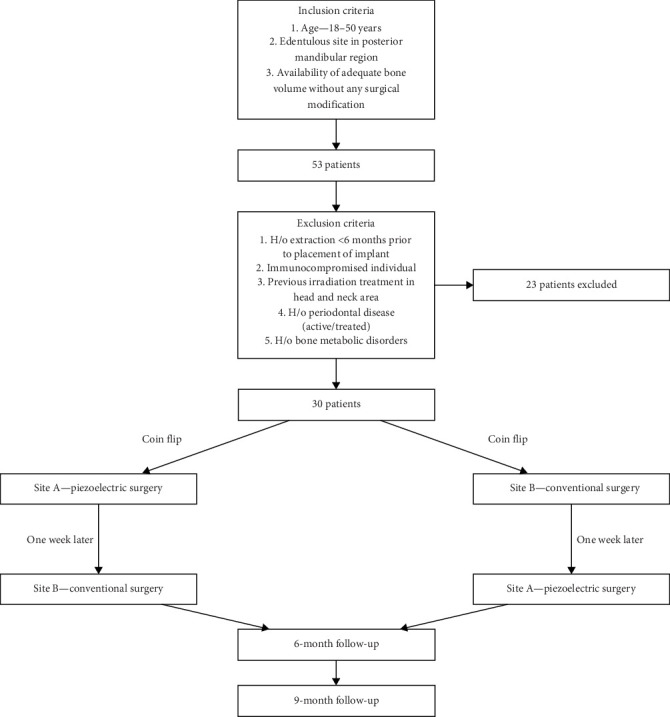
Patient inclusion/exclusion criteria and distribution into surgical groups. Inclusion criteria: Participants aged 18–50 years with edentulous posterior mandibular sites and sufficient bone volume without surgical modification requirements. Exclusion criteria: Participants with an extraction history less than 6 months prior to implant placement, immunocompromised status, previous head and neck irradiation, active or treated periodontal disease, or bone metabolic disorders.

The sample size was determined using G^*⁣*^*∗*^^Power 3.1.9.2 software, with an effect size of 0.5, a power of 80%, and a significance level of 5% in the study groups, keeping ISQ values as the primary outcome. The analysis indicated that a minimum of 27 participants per group was necessary. Given the split-mouth design and to accommodate potential dropouts, a total sample size of 30 patients was finalized. A total of 53 patients were consecutively recruited for this split-mouth clinical and radiological study, which was approved by the ethics department at the institution. Each patient had at least one edentulous site present bilaterally in the posterior mandibular region, ensuring comparability of sites; however, based on the exclusion criteria mentioned herewith, 23 were eventually excluded (Figure 1). Each patient had two surgical sites, A and B, which received piezoelectric surgery and conventional surgery for comparison.

### 2.2. Inclusion/Exclusion Criteria

Patients were selected based on the following inclusion criteria: age between 18 and 50 years, presence of an edentulous site in the posterior mandibular region, and availability of adequate bone volume without requiring any surgical modification. Exclusion criteria included a history of extraction less than 6 months prior to implant placement, immunocompromised status, systemic disease, previous irradiation treatment in the head and neck area, history of active or treated periodontal disease, and history of bone metabolic disorders. Smokers were excluded. The full details of the inclusion and exclusion criteria are presented in Figure 1.

### 2.3. Grouping

Following patient selection, patients were allocated to two groups based on the type of implant Site A preparation: Site A, where implant placement was conducted using piezoelectric surgery, and Site B, where conventional surgery was employed for implant placement. The randomization of bilateral edentulous sites for implant placement was determined using a coin flip. Since the washout period between the two interventions was 1 week, the assignment order of the treatment was randomized to remove the influence of the treatment on the outcome. Allocation concealment was achieved using sequentially numbered opaque sealed envelopes. This was a double-blind study where the patients and the outcome assessor not participating in the surgical procedure were blinded. The detailed study schedule is shown in Figure 1. Prior to any procedure, all patients were thoroughly informed of the study details, and written informed consent was obtained. The study protocol was approved by the ethics committee of the institute.

### 2.4. Presurgical Evaluation

**Figure 2:**
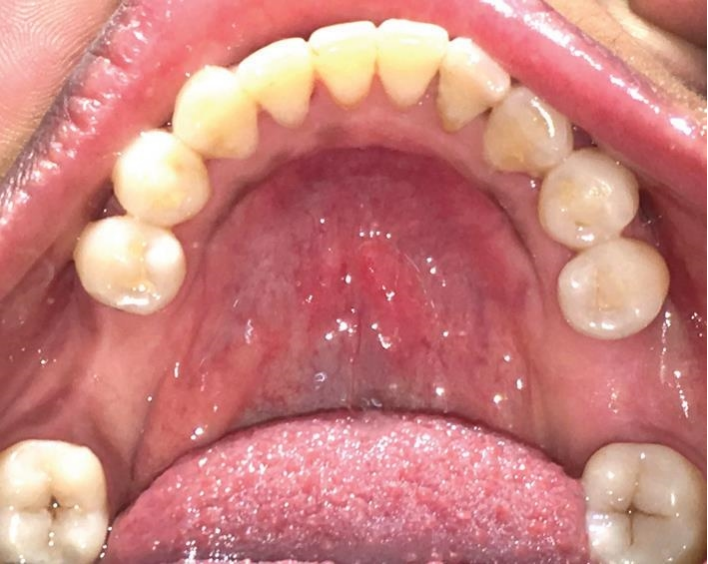
Preoperative picture depicting case with bilateral edentulous site in mandibular posterior region.

During the initial visit, a comprehensive presurgical evaluation was conducted, which included recording the patient's case history and performing a thorough clinical examination, encompassing both intra- and extra-oral assessments (Figure 2). Maxillary and mandibular impressions were obtained using alginate impression material and stock trays, followed by pouring the impressions using a Type III Dental Stone to obtain diagnostic casts. Radiographic evaluation was performed using digital RVG and panoramic radiography. Standardization of radiographs was ensured using digital RVG (DBSWIN Durr Dental Image) with a Rinn XCP film holder and custom-made occlusal splint. Additionally, cone-beam computed tomography (CBCT) scans were used for bone assessment in preparation for implant placement. Presurgical blood investigations were carried out, including complete blood count (CBC), INR, blood sugar (fasting and PP), and routine urine examination.

### 2.5. Surgical Procedure for Implant Placement

Surgical osteotomies were conducted to place cylindrical, screw-type, bone-level implants sandblasted with calcium sulfate and acid etched surface, deep internal hex connection, and platform-switched design (A'B I5 dental implant system). All surgical procedures were performed freehand without the use of surgical guides by a single operator (Manish Rathi) at both sites.

**Figure 3:**
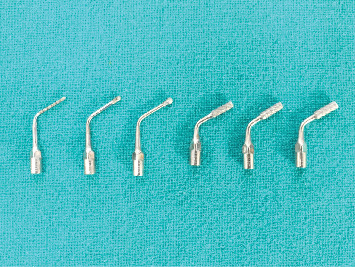
Piezosurgical inserts used for implant site osteotomy.

**Figure 4:**
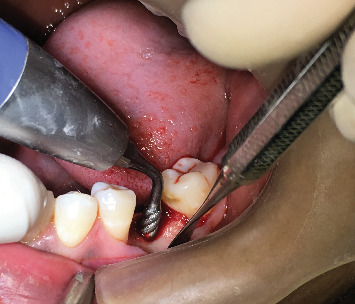
Site A piezosurgical implant site preparation using piezosurgical inserts.

At Site A, implant insertion was performed using piezoelectric surgery under local anesthesia. A paracrestal incision was made toward the lingual side, followed by raising a full-thickness mucoperiosteal flap toward the buccal side. Sequential osteotomy was carried out using EMS piezo inserts (Piezonmaster, EMS SA, Nyon, Switzerland) (Figure 3), gradually preparing the site with increasing diameters from MB1 to MB6 at a frequency of 30 kHz with an amplitude of 60–110 mm and power of 5 W, with physiological solution (0.9% normal saline) as a coolant (Figure 4). Following site preparation, implants were inserted until the crestal level, and primary stability was evaluated using MulTipeg inserted into the implant and resonance frequency analysis (RFA) scanner (Penguin RFA, Integration Diagnostics Sweden AB) directed toward the magnet on the top of the MulTipeg. Flap closure was then performed using simple interrupted sutures.

**Figure 5:**
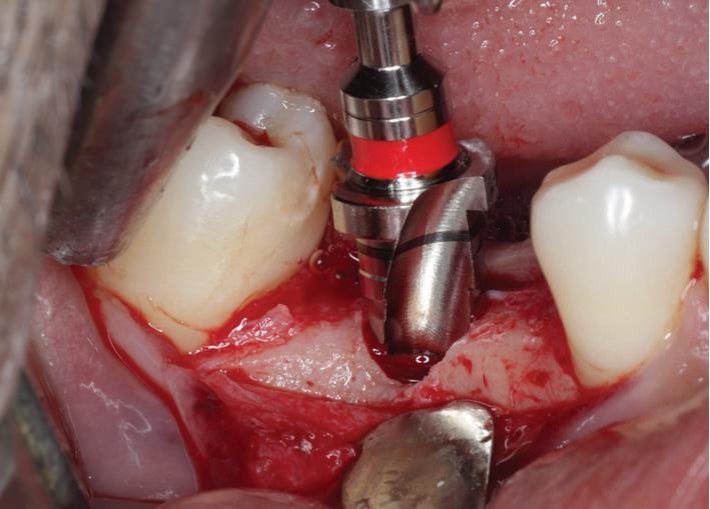
Site B conventional implant site preparation using rotary drills.

At Site B, implant insertion was conducted using the conventional rotary technique under local anesthesia (Figure 5). Like Site A, paracrestal incision was made, and a mucoperiosteal flap was raised. Sequential osteotomy was performed using conventional drill bits at 800 rpm with physiological solution (0.9% normal saline) as a coolant, gradually increasing the diameter to one size smaller than the corresponding implant diameter. Implants were inserted to the crestal level, and primary stability was assessed using an RFA scanner. Flap closure was completed using simple interrupted sutures.

### 2.6. Postoperative Care

Following implant placement, patients were prescribed antibiotics (Amoxiclav 1 g bd) and analgesics (Ibuprofen 400 mg tid) for 5 days, along with 0.12% Chlorhexidine Digluconate mouthrinse for 2 weeks. Oral hygiene instructions were provided, and patients were given a visual analog scale (VAS) to record postoperative discomfort. Suture removal was scheduled for the 7th day postsurgery. All patients were scheduled for a follow-up review after 6 months.

### 2.7. Postoperative Follow-Up

Patient assessments at 6 and 9 months postoperatively for dental implants are reasonable for comparing results between piezoelectric surgery and traditional surgery. Both bone density and stability vary significantly over this period [10, 14, 30]. Evaluating bone density at 6 and 9 months is especially useful since bone remodeling and healing processes continue for a long time after surgery in humans [30]. Previous studies have investigated bone density in response to piezoelectric surgery using animal models with appropriately shortened timelines; however, this study addressed piezoelectric surgery in people, requiring extended timelines for which 6 and 9 months are appropriate [30]. Implant stability has also been assessed at up to 1 year postsurgery [14].

### 2.8. Stage II Surgery (Implant Exposure)

**Figure 6:**
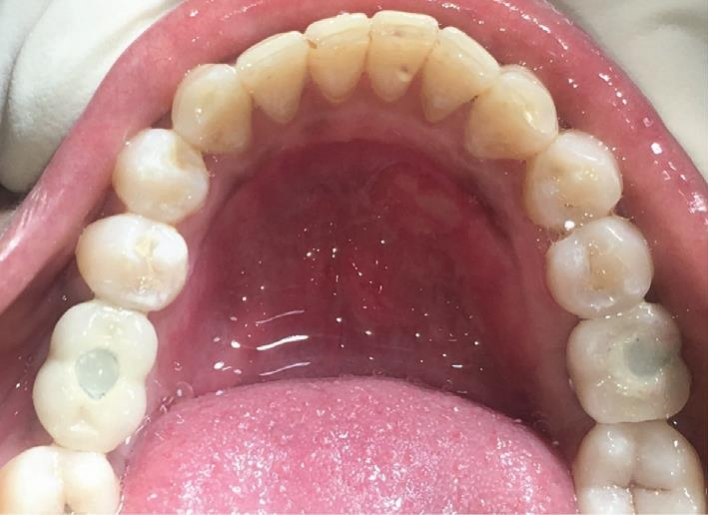
Postrestorative occlusal view showing bilateral implant-supported single tooth prosthesis in mandibular posterior region.

After a 6-month osseointegration period, Stage II implant surgery was performed. Under local anesthesia, a full-thickness mucoperiosteal flap was raised, and implants were exposed using the split-finger technique. Implant stability was measured using an RFA scanner, and corresponding healing abutments were inserted. Flaps were sutured around the abutments, and sutures were removed after 7 days. Implant loading was completed after 1 month of implant exposure (Figure 6).

### 2.9. Postsurgical Evaluation

At 6 and 9 months postsurgery, bone density values were reassessed using dual-energy X-ray absorptiometry (DEXA). Implant stability was measured during Stage II surgery using an RFA analyzer. Radiographic evaluations were performed, and clinical parameters, including peri-implant mucosal parameters and pain assessment, were recorded.

The VAS is a popular instrument in pain research for measuring the severity of pain felt by individuals. It usually consists of a straight line, ~10 cm long, with endpoints representing the extreme boundaries of the pain scale. One end of the line indicates “no pain,” while the other represents the “worst imaginable pain.” Participants were asked to mark a point on the line that represented their current pain severity. The VAS score was then calculated as the distance from the “no pain” endpoint to the designated point, resulting in a continuous pain scale on the seventh day postoperatively. This approach is favorable because of its simplicity and sensitivity to changes in pain levels, making it an important tool for evaluating pain interventions and therapies in both clinical and research contexts [31].

### 2.10. Statistical Analysis

All statistical analysis was performed with R version 4.2.2 Patched (2022-11-10 r83330)—“Innocent and Trusting” Copyright (C) 2022 The R Foundation for Statistical Computing, Vienna, Austria. These analyses included derivation of the mean, median, standard deviation (SD), and 95% confidence interval (CI). The Shapiro–Wilk test, data transformations, one-sample *t* tests, and paired *t* tests were performed to compare the means of the samples. A one-sample proportion test was used to test assumptions about age distribution. The mean age of the Indian population was assumed to be 28. The typical length of the dental implants was assumed to be 10 mm.

## 3. Results

Table 1 presents findings regarding patient demographics, bone width, and implant sizes across the two surgical sites. Thirty patients were included in the study, with a mean age of 35.7 years (SD = 7.1), significantly different from the hypothesized population mean of India, which is 28 years (*p*  < 0.001, *⁣*^*∗∗∗*^). There was no significant difference in sex distribution (*p*=0.100, NS), with 10 females (33.3%) in the sample. Bone width at Site A (piezoelectric surgery) averaged 5.863 mm (SD = 0.2), significantly narrower than conventional surgery at Site B (mean = 6.087 mm, SD = 0.5; *p*=0.017, *⁣*^*∗*^). Implant sizes with a fixed length of 10 mm differed significantly in diameter between the sites; despite a median diameter of 3.5 mm at both sites, Site B had, on average, implant sizes 2.75 mm wider in diameter. To address this statistical discrepancy, the Bonferroni correction is applied, adjusting the *p*-value to 0.0167 by dividing 0.05 by 3 for the comparisons between the two groups. The threshold for statistical significance was thus established at 0.0167.

No adverse events occurred during the study.  Table 2 presents the bone density measurements (g/cm^2^) over time at both surgical sites. At baseline, the bone density did not differ significantly between the sites. However, at 6 months, Site A showed a slight decrease in bone density (mean = 1.366 g/cm^2^) compared to Site B (mean = 1.331 g/cm^2^), although this difference was not statistically significant (*p*=0.4612, NS). At 9 months, Site A exhibited a slight increase in bone density (mean = 1.404 g/cm^2^), which was higher than that at Site B (mean = 1.330 g/cm^2^; *p*=0.035,) which was statistically nonsignificant.

Table 3 shows the ISQ values over time at both surgical sites. At baseline, there was no significant difference in ISQ values between the sites. However, at 6 months, Site A (piezoelectric surgery) demonstrated significantly higher ISQ values (mean = 76.7) than Site B (mean = 72.8; *p* ≤ 0.001). While the difference in ISQ at 6 months achieved statistical significance (*p* ≤ 0.001), the absolute difference of 0.56 ISQ is unlikely to hold clinical significance.

Table 4 presents the findings on pain perception measured by the VAS for both surgical techniques. Patients who underwent conventional surgery reported significantly higher pain levels (mean = 7.2) than those who underwent piezoelectric surgery (mean = 5.9; *p* ≤ 0.001, *⁣*^*∗∗∗*^). This indicates that piezoelectric surgery may provide benefits in terms of patient-reported outcomes related to pain when compared to conventional methods.

## 4. Discussion

Advancements in dental implantology have significantly contributed to improving patient care and treatment outcomes, particularly in addressing issues related to edentulism. The introduction of dental implants has revolutionized the field by offering restoration solutions for complete or partial edentulism, thus enhancing the overall quality of life of patients. However, the success of dental implants relies heavily on osseointegration, implant stability, and peri-implant tissue parameters.

The comparative evaluation conducted in this study between piezoelectric surgery and conventional surgery in implant site preparation sheds light on important aspects of implantology. The findings revealed significant differences between the two techniques in terms of patient-reported outcomes.

There was a greater increase in bone density at 9 months compared to the control in this study; however, it was not clinically significant. The primary importance of this result is that it has been demonstrated in humans. Previous studies have only addressed bone density in animal models [32, 33]. Furthermore, due to the differences in bone morphology between humans and small animal models, the timeline was necessarily extended, showing that the results of piezoelectric surgery are long-term and enduring in humans.

The findings of the present study are in line with the previous work that found no difference between conventional surgery and piezoelectric surgery in terms of implant stability [14, 16]. One possible explanation for this is the fact that previous studies have shown implant stability to converge over time when comparing the piezoelectric surgery group to others. Due to the extended timelines employed by this study to monitor bone density, it is possible that earlier differences in stability were lost to our analysis. One of the three studies that previously also found no significant difference in stability also measured stability at an extended time point of 1 year, which is greater than any time points used in this study [14].

This study adds to the body of literature that suggests that patients experience less pain immediately following surgery using piezoelectric tools compared to conventional surgical drills [24–28]. The bulk of research supports this, and our work adds to the evidence. The exact reason why some studies find the opposite to be true is unclear [21, 22]. The variations in patient discomfort are not large in magnitude, and the discrepancy across trials might be attributed to the dental surgeon's skill level and surgical method [34].

Additionally, this technique generates lower temperatures compared to conventional methods and avoids the production of excess force that can lead to microcracks in the bone. Such innovations potentially enhance osteoblastic activity by minimizing damage and thermal necrosis during procedures.

Another important study which compared piezoelectric surgery with conventional osteotomy showed that piezoelectric surgery may modify and reduce bone-destructive inflammatory responses during implant osseointegration, as evidenced by lower RANKL levels, this molecular-level advantage did not translate into significant differences in crestal bone loss, suggesting that might be a less traumatic osteotomy modality than drilling on a cellular level, but this benefit may not be clinically apparent in terms of bone preservation in the short term. The study also provides novel data on the changes in the RANKL-OPG system during osseointegration, indicating its role as a key bone remodeling mechanism in the process of implant integration. These findings highlight the complex interplay between molecular processes and clinical outcomes in dental implantology, emphasizing the need for further research to fully understand and optimize implant placement techniques [35].

A systematic review and meta-analysis found no significant differences in primary stability (measured by ISQ) between implants placed using piezoelectric surgery versus conventional drilling. At 3 months postimplantation, implants placed using piezoelectric surgery demonstrated significantly higher secondary stability [36]. The present study observed a similar pattern, with secondary stability being statistically significant in favor of the piezosurgical approach.

Piezoelectric surgery utilizing ultrasonic oscillations for precise cutting demonstrated potential advantages over conventional techniques in our study. Notably, it exhibits higher bone density and lower postoperative discomfort, indicating its potential to enhance clinical outcomes and patient satisfaction. A strength of this study is its methodology, including the selection and grouping of subjects based on rigorous inclusion and exclusion criteria, which maintains the reliability and validity of the findings. The utilization of comprehensive presurgical evaluations, including radiographic assessments and blood investigations, adds to the thoroughness of this research approach.

Furthermore, mid-term follow-up evaluations at 6- and 9-month postsurgery provide valuable insights into the durability and sustainability of the outcomes. The assessment of bone density and implant stability over time offers a comprehensive understanding of the treatment's efficacy and potential complications.

The limitations of the study include the relatively small sample size and the absence of standardized procedures for reporting the surgeon's technique, such as time to completion and use of irrigation fluid. Other limitations include the limited washout period between two interventions and significant difference between bone width and implant size in test group, which was, however, adjusted statistically. Also, the baseline pain perception of the patients could have been standardized in order to derive more meaningful conclusions in terms of pain perception. Additionally, the comparison between piezoelectric surgery and conventional surgery could benefit from further investigation into factors such as inflammation and esthetic outcomes. In addition to assessments at 6 and 9 months, the study may have benefited from earlier assessments at perhaps 30 and 90 days in order to make the results more comparable with previous literature.

## 5. Conclusions

In conclusion, this study found that piezoelectric surgery offers potential benefits over conventional surgery in terms of mid-term bone density and patient-perceived pain, but that there was little difference in implant stability between the two techniques. By providing valuable insights into the comparative effectiveness of piezoelectric surgery and conventional surgery, this research contributes to optimizing treatment protocols and improving patient care in implant dentistry.

## Figures and Tables

**Table 1:** Demographic and anatomical characteristics of study cohort.

Categories	Value(s)	Difference	*p*-Value	Sig. level
*n* (patients)	30	—	N/A	—
Age ∈ (18, 50) (years)				
Mean, *μ*₀ = 28	35.7	7.7	<0.001	*⁣* ^*∗∗∗*^
Range	24–49	—	—	—
SD	7.1	—	—	—
95% CI	33.1–38.3	—	—	—
Female sex, $\hat{p}$ = 0.5				
*n* (%)	10 (33.3)	−16.70%	0.100	NS
95% CI (%)	17.9–52.9	—	—	—
Bone width Site A (mm)				
Mean	5.863	−0.223	0.017	*⁣* ^*∗*^
Range	5.3–6.4	—	—	—
SD	0.2	—	—	—
95% CI, *μ*₀ = 5.975	5.776–5.950	—	—	—
Bone width Site B (mm)				
Mean	6.087	0.223	0.017	*⁣* ^*∗*^
Range	5.3–7.1	—	—	—
SD	0.5	—	—	—
95% CI, *μ*₀ = 5.975	5.892–6.281	—	—	—
Implant diameter Site A (mm)				
Median	3.5	−2.75	<0.001	*⁣* ^*∗∗∗*^
Range	3.3–3.5	—	—	—
IQR	0	—	—	—
95% CI, *μ*₀ = 8	3.468–3.506	—	—	—
Implant diameter Site B (mm)				
Median	3.5	2.75	<0.001	*⁣* ^*∗∗∗*^
Range	3.3–4.5	—	—	—
IQR	0.65	—	—	—
95% CI, *μ*₀ = 8	3.622–3.901	—	—	—

*Note:* This includes age distribution, sex distribution, bone width at two sites (A and B), and implant size at the same sites. Statistical analyses reveal significant differences in age distribution, with a mean age of 35.7 years (*p* < 0.001), as well as in bone width at both Sites A and B (*p*=0.017). There were also significant differences in implant size at both sites (*p* < 0.001), with medians of 3.5 mm. “NS” denotes nonsignificance, “*⁣*^*∗*^*p* < 0.05,” and “*⁣*^*∗∗∗*^*p* < 0.001.”

**Table 2:** Bone density over time at surgical sites.

Site A (piezoelectric surgery)					Site B (conventional surgery)				Statistical significance		
	Value(s)	Δ *bd*	*p* -Value	Sig. level	Value(s)	Δ *bd*	*p* -Value	Sig. level	Δ (Δ *bd*)	*p* -Value	Sig. level
Baseline (g/cm^2^)											
Mean	1.393	N/A	N/A	N/A	1.355	N/A	N/A	N/A	N/A	N/A	N/A
Range	1.060– 2.210	—	—	—	1.060– 1.730	—	—	—	—	—	—
SD	0.254	—	—	—	0.209	—	—	—	—	—	—
95% CI, *μ*₀ = 1.374	1.299–1.488	—	—	—	1.277–1.433	—	—	—	—	—	—
6 months (g/cm^2^)											
Mean	1.366	−0.027	0.028	*⁣* ^*∗*^	1.331	−0.023	0.4612	NS	−0.004	0.906	NS
Range	1.020–2.090	—	—	—	1.010–1.980	—	—	—	—	—	—
SD	0.235	—	—	—	0.233	—	—	—	—	—	—
95% CI, *μ*₀ = 1.374	1.278–1.454	—	—	—	1.244–1.418	—	—	—	—	—	—
9 months (g/cm^2^)											
Mean	1.404	0.011	0.228	NS	1.330	−0.025	0.035	*⁣* ^*∗*^	0.035	0.001	*⁣* ^*∗∗∗*^
Range	1.090–2.150	—	—	—	1.030–1.670	—	—	—	—	—	—
SD	0.239	—	—	—	0.201	—	—	—	—	—	—
95% CI, *μ*₀ = 1.374	1.315–1.493	—	—	—	1.255–1.405	—	—	—	—	—	—

*Note:* This table compares bone density changes between piezoelectric surgery (Site A) and conventional surgery (Site B) at baseline, 6-month, and 9-month postsurgery. At baseline, mean bone density was 1.393 g/cm^2^ (Site A) and 1.355 g/cm^2^ (Site B), with no significant difference. At 6 months, bone density decreased slightly in both groups, with a mean decrease of 0.027 g/cm^2^ (*p*=0.028) for Site A and 0.023 g/cm^2^ (*p*=0.4612) for Site B. At 9 months, Site A showed a slight increase in bone density (mean increase of 0.011 g/cm^2^, *p*=0.228), while Site B exhibited a significant decrease (mean decrease of 0.025 g/cm^2^, *p*=0.035). The difference in bone density changes between the two sites was statistically significant at 9 months (Δ [Δ *bd*] = 0.035 g/cm^2^, *p*=0.001). “NS” denotes nonsignificance, “*⁣*^*∗*^*p* < 0.05,” and “*⁣*^*∗∗∗*^*p* < 0.001.”

**Table 3:** Implant stability quotient (ISQ) values over time at surgical sites.

Site A (piezoelectric surgery)					Site B (conventional surgery)				Statistical significance		
	Value(s)	Δ Stability	*p*-Value	Sig. level	Value(s)	Δ Stability	*p*-Value	Sig. level	Δ (Δ Stability)	*p*-Value	Sig. level
Baseline (ISQ)											
Mean	74.3	N/A	N/A	N/A	70.9	N/A	N/A	N/A	N/A	N/A	N/A
Range	61.0–81.0	—	—	—	61.0–84.0	—	—	—	—	—	—
SD	4.178	—	—	—	5.024	—	—	—	—	—	—
95% CI, *μ*₀ = 70	72.740– 75.860	—	—	—	69.057–72.809	—	—	—	—	—	—
6 months (ISQ)											
Mean	76.7	2.400	<0.001	*⁣* ^*∗∗∗*^	72.8	1.840	0.001	*⁣* ^*∗∗*^	0.560	0.509	NS
Range	63.0–82.0	—	—	—	61.0–79.0	—	—	—	—	—	—
SD	3.958	—	—	—	4.133	—	—	—	—	—	—
95% CI, *μ*₀ = 70	75.222– 78.178	—	—	—	71.223–74.310	—	—	—	—	—	—

*Note:* This table compares stability changes between piezoelectric surgery (Site A) and conventional surgery (Site B) as measured by implant stability quotient (ISQ) at baseline and 6-month postsurgery. At baseline, mean ISQ was 74.3 for Site A and 70.9 for Site B, with no significant difference. At 6 months, both sites showed an increase in stability, with Site A increasing by 2.400 (*p* < 0.001) and Site B by 1.840 (*p*=0.001). The difference in stability changes between the two sites was not statistically significant (Δ [Δ stability] = 0.560, *p*=0.509, NS). “NS” denotes nonsignificance, “*p* < 0.01,” and “*p* < 0.001.”

**Table 4:** Pain perception following different surgical techniques.

Site	Value(s)	Δ VAS	*p*-Value	Sig. level
VAS (piezoelectric surgery)				
Mean	5.9	N/A	N/A	N/A
Range	3.0–9.0	—	—	—
SD	1.373	—	—	—
95% CI, *μ*₀ = 0	5.387–6.413	—	—	—
VAS (conventional surgery)				
Mean	7.2	1.300	<0.001	*⁣* ^*∗∗∗*^
Range	5.0–9.0	—	—	—
SD	1.270	—	—	—
95% CI, *μ*₀ = 0	6.726–7.674	—	—	—

*Note:* This table compares the visual analog scale (VAS) scores between piezoelectric surgery and conventional surgery. The VAS scores represent pain perception, with higher scores indicating higher pain levels. At baseline, the mean VAS score for piezoelectric surgery was 5.9, with a range of 3.0–9.0, while for conventional surgery, the mean VAS score was 7.2, with a range of 5.0–9.0. The difference in mean VAS scores between the two groups was statistically significant (*p* < 0.001). “N/A” indicates not applicable. The 95% confidence intervals for the mean VAS scores are provided. “*p* < 0.001.”

## Data Availability

The data are available from the corresponding author.

## General Statement

The authors have adhered to the CONSORT Checklist.
